# Disentangling the Contributions of Plant Taxonomic and Functional Diversities in Shaping Aboveground Biomass of a Restored Forest Landscape in Southern China

**DOI:** 10.3390/plants8120612

**Published:** 2019-12-16

**Authors:** Md. Abu Hanif, Qingshui Yu, Xingquan Rao, Weijun Shen

**Affiliations:** 1 Key Laboratory of Vegetation Restoration and Management of Degraded Ecosystem, South China Botanical Garden, Chinese Academy of Sciences, Guangzhou 510650, China; hanif_hstu@yahoo.com (M.A.H.); yuqingshui@pku.edu.cn (Q.Y.); rxq99@scbg.ac.cn (X.R.); 2 University of Chinese Academy of Sciences, Beijing 100000, China; 3 Department of Agroforestry and Environment, Hajee Mohammad Danesh Science and Technology University, Dinajpur-5200, Bangladesh; 4 Department of Ecology, College of urban and Environmental Sciences, Key Laboratory for Earth Surface Processes of the Ministry of Education, Peking University, Beijing 100871, China

**Keywords:** plant functional diversity, plant taxonomic diversity, biodiversity-ecosystem functioning, soil nutrients, ecological restoration

## Abstract

Restoration is essential for supporting key ecosystem functions such as aboveground biomass production. However, the relative importance of functional versus taxonomic diversity in predicting aboveground biomass during restoration is poorly studied. Here, we used a trait-based approach to test for the importance of multiple plant diversity attributes in regulating aboveground biomass in a 30-years-old restored subtropical forest in southern China. We show that both taxonomic and functional diversities are significant and positive regulators of aboveground biomass; however, functional diversity (FD) was more important than taxonomic diversity (species richness) in controlling aboveground biomass. FD had the strongest direct effect on aboveground biomass compared with species richness, soil nutrients, and community weighted mean (CWM) traits. Our results further indicate that leaf and root morphological traits and traits related to the nutrient content in plant tissues represent the existence of a leaf and root economic spectrum, and the acquisitive resource use strategy influenced aboveground biomass. Our results suggest that both taxonomic and FD play a role in shaping aboveground biomass, but FD is more important in supporting aboveground biomass in this type of environments. These results imply that enhancing FD is important to restoring and managing degraded forest landscapes.

## 1. Introduction

Forest ecosystems are essential for capturing atmospheric carbon, which is then deposited in above- and below-ground biomass [1]. The woody bole and branches where a substantial portion of atmospheric carbon is stored form this aboveground biomass [2]. Landscape-level distribution of aboveground biomass has been well documented [3] but the fundamental mechanisms of producing and retaining aboveground biomass, particularly in restored forest ecosystems is less explored. Logically, aboveground biomass is expected to increase during secondary succession [4] but the time required to reach the maximum level of aboveground biomass might vary across forest ecosystems [5]. The variation of aboveground biomass might be influenced by several factors including environmental variables such as soil properties and forest community characteristics such as plant taxonomic diversity (TD) and functional diversity. Functional diversity is defined as the distribution and relative abundance of functional traits in a community [6]. Functional dispersion is one of the multidimensional indexes of FD which captures the spread of species traits from the centroid. It has been used in this article to represent FD. However, our understanding of the relative importance of functional and taxonomic diversity in affecting aboveground biomass in restored woody forest ecosystems as opposed to the herbaceous grassland ecosystem is relatively understudied.

**Figure 1:**
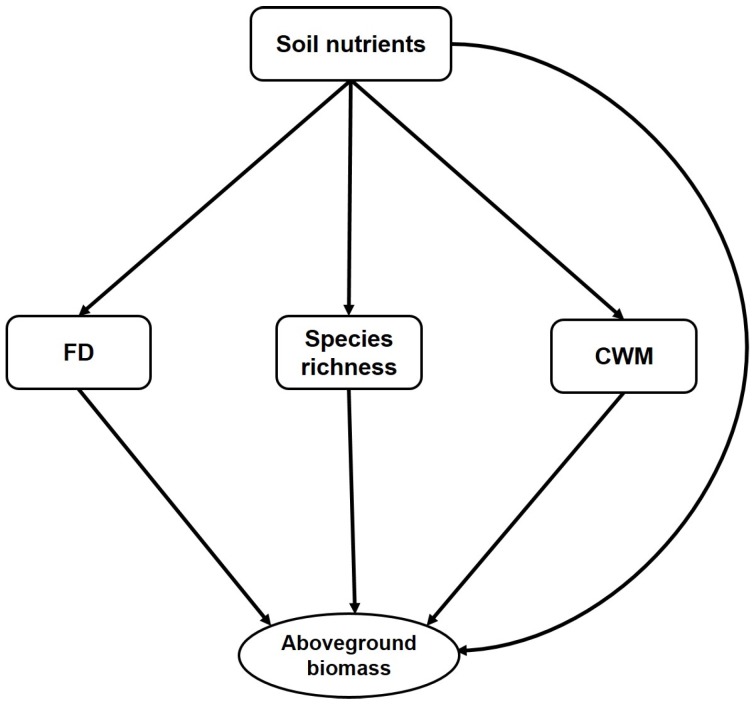
A conceptual model for linking the multivariate relationships among soil nutrient, species richness, functional diversity, community weighted mean (CWM) traits, and the aboveground biomass of a restored forest landscape in southern China. Soil nutrients were characterized by the principal component analysis (PCA) scores of major soil nutrients, functional diversity was characterized by the functional divergence of 15 traits and CWM was characterized by PCA scores of all the traits from above-and below-ground. Each hypothesized path has been discussed in the introduction section.

Taxonomic diversity and plant functional diversity have linkage with aboveground biomass and soil properties [7,8]. Species richness has been positively linked with aboveground biomass from strata (understorey and overstorey) to the whole community [7]. Moreover, species richness explained 28.5% of the variation in the total carbon stock in a subtropical forest ecosystem in southeastern China [9]. Species richness creates more niche space and improves efficient nutrient utilization, therefore increases aboveground biomass [10]. According to our hypothesis (Figure 1), the relationship between aboveground biomass and plant functional diversity fundamentally relies on species richness and soil nutrients. Functional diversity has important linkages with ecosystem processes. Functional diversity has been used for describing the variability in carbon stock deposited in the aboveground biomass [11]. The relationship between carbon accumulation in the aboveground biomass and plant functional diversity depends upon the forest types and the diversity of functional traits in a plant community [1,12]. A higher diversity of traits associated with resource acquisition creates niche complementarity which allows the community to have greater access to the entire resource pool [6]. Therefore, functional diversity should have a positive correlation with aboveground biomass when resource complementarity is the underlying driver. However, if the community has a higher abundance of productive species, then species richness would have a stronger relationship with aboveground biomass than functional diversity [1].

There are two important hypotheses by which we can characterize the effect of functional diversity on aboveground biomass. The mass ratio hypothesis states that dominant species and their traits have a relatively stronger effect on ecosystem processes than do functionally diverse rare species [13]. The mass ratio or community weighted mean (CWM) is the trait value of the species weighted by the species relative abundance in a community [14,15]. The niche complementary hypothesis posits that higher aboveground biomass in a forest community is regulated by diverse species that create more available niche space and the availability of diverse resources ensuring proper utilization of the resources [12,16,17]. According to our hypothesis (Figure 1), functional diversity, species richness, and CWM traits will correlate with and contribute to aboveground biomass. Previous empirical studies showed evidence of CWM and functional diversity alone [18,19] or jointly [12] influencing aboveground biomass. Additionally, ecosystem processes can be directly influenced by soil properties by altering the ecosystem flux rates of matter and energy [20]. Soil nutrients were also found to influence aboveground biomass indirectly through species richness and plant functional traits in tropical forests [6,20]. Therefore, the conceptual framework (Figure 1) of this study postulates that soil nutrients will directly influence aboveground biomass and that species richness and plant functional diversity will do so indirectly. Previously we found that the taxonomic and phylogenetic diversity of the studied forest has been increased steadily during restoration, and deterministic and stochastic processes drove community composition of the forest [21]. Former studies in other regions of the globe focused on the plant trait and ecosystem functioning linkages in artificially managed plant communities or environmental gradients. In these communities, functional diversity would be very low compared to under natural conditions. Furthermore, along environmental gradients, it is very difficult to differentiate the effects of plant communities on ecosystem functions and soil properties. Therefore, in realistic communities, the linkages between plant traits and ecosystem functions need to be explored [22].

Plant functional traits have a direct and indirect effect on plant fitness and productivity [23]. Aboveground biomass might be mechanistically correlated with the plant traits of a community related to the growth rate, and the ability to capture, store, and release carbon and resources [19]. Important ecosystem functions such as nutrient cycling, respiration, and productivity were found to be strongly influenced by specific leaf area (SLA) and the leaf nitrogen content (LN) [24]. SLA, LN, and the leaf dry matter content (LDMC) have been found to have a positive relationship with aboveground biomass [6,12,18]. Moreover, it is important to identify key plant functional traits, both above- and below-ground, which can be used to predict individual or multiple functions of the ecosystem. Previous studies mainly focused on aboveground plant traits as the variables of plant functional diversity, but ecosystem functions can be directly influenced by root traits, so these need to be incorporated in the study of the biodiversity-ecosystem functioning relationship [25]. Root traits have wider implications for soil processes likely to impact ecosystem functioning [26,27] and have been rarely studied.

Herein, we aim to decipher the relationship between biodiversity metrics (taxonomic and functional diversity) and ecosystem function (aboveground biomass) in a restored forest landscape in subtropical China. The forest landscape consists of four plantation types: an *Acacia mangium* (AM) plantation, a mixed *Eucalyptus* (EE) species plantation, a mixed coniferous species (MC) plantation, and a mixed native species (NS) plantation. We address the following questions: i) Which biodiversity metrics (taxonomic diversity or functional diversity) is the more important regulator of aboveground biomass? ii) What are the important plant functional traits driving aboveground biomass?

## 2. Results

The results of the Principal Component Analysis (PCA) of 15 functional traits (CWM) and 4 soil properties are shown in Appendix Table A1. The first PC axis for CWM traits explained 91.44% of overall variability and the second PC axis accounted for 6.71% of overall variability (Table A1). The first PC axis dominated by traits related to statute, morphological and elemental concentration in leaf and roots (Table A1). In case of soil PCA, the first PC axis which explained 66.05% of overall variability, which mainly represented the variability in soil total carbon (SOC) and soil total nitrogen (TN). The second soil PCA axis, which explained 33.82% overall variance, mainly represented the variability in total phosphorus content (TP) and total potassium content (TK) in soil (Table A1).

**Figure 2:**
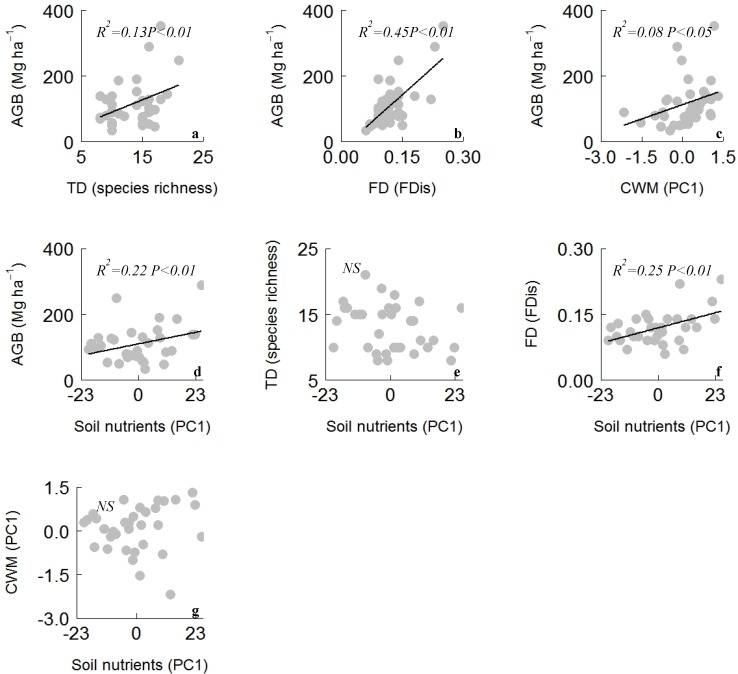
Relationship between above ground biomass and (**a**) taxonomic diversity (species richness), (**b**) functional diversity (FDis), (**c**) community weighted mean (CWM principal component—PC1) traits, and (**d**) soil nutrients (PC1). Relationship between soil nutrients and (**e**) taxonomic diversity (species richness), (**f**) functional diversity (FDis), (**g**) community weighted mean (CWM PC1) traits in the restored forest ecosystem. The black line represents the fitted linear regression. Significant *p* < 0.05 and nonsignificant (NS) *p* > 0.05.

The ordinary least squares (OLS) linear regression analysis showed that aboveground biomass was significantly (*p* < 0.01) and positively associated with TD and FD (Figure 2a,b). The relationship of aboveground biomass with FD (R^2^ = 0.45) was stronger than that with TD (R^2^ = 0.13). FD (R^2^ = 0.45 vs. 0.13) explained more variations in aboveground biomass than did TD. Aboveground biomass was also significantly related to CWM (R^2^ = 0.08) traits (Figure 2c) and soil nutrients (R^2^ = 0.22) (Figure 2d). Soil nutrients had a significant (*p* < 0.01) relationship with FD and nonsignificant (*p* > 0.05) relationship with TD and CWM traits (Figure 2e,f). The analysis of covariance (ANCOVA) showed that plantations types had an influence on aboveground biomass (AGB) and TD bivariate relationships, while the other bivariate relationship between AGB and predictor variables was not influenced by plantation types (Table A2).

**Figure 3:**
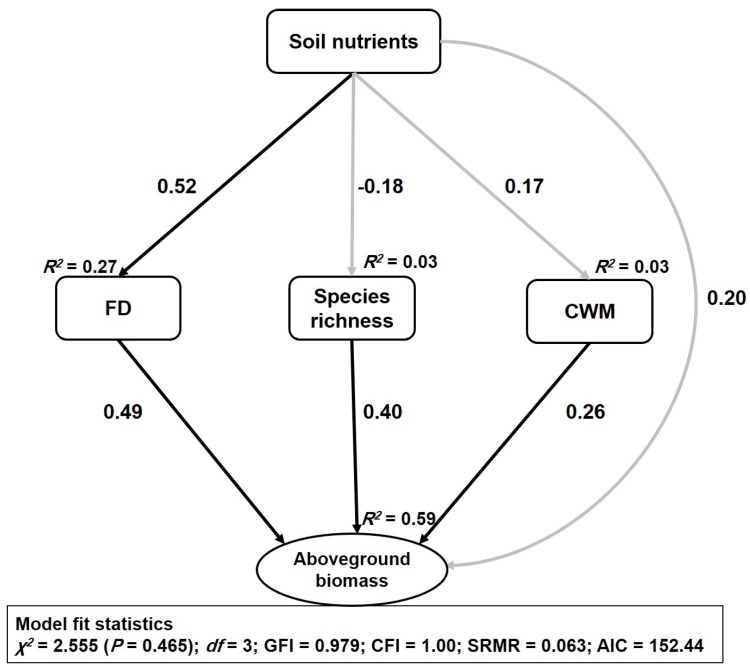
The structural equation modelling showed how soil nutrients, taxonomic diversity (species richness), functional diversity (FD), and community weighted mean traits (CWM traits) drive above-ground biomass. Effect size of relationship was presented through the numbers adjacent to the arrows. Solid black arrows indicate a positive significant relationship, and grey arrows indicates as non-significant relationship. Goodness-fit statistics for model are as follows: χ2 = 2.555(*p* = 0.465), d.f. = 3; GFI = 0.979; CFI = 1.00; SRMR = 0.063, AIC = 152.44.

Structural equation modelling (SEM) accounted for 59% of the variation in aboveground biomass (Figure 3). FD had the strongest positive direct relationship with aboveground biomass (β = 0.49, *p* < 0.001), followed by species richness (β = 0.40, *p* < 0.001) and CWM traits (β = 0.26, *p* < 0.01). The direct effect of soil nutrients on aboveground biomass was found to be nonsignificant (β = 0.20, *p* > 0.05), but the indirect effect of soil nutrients through FD was found to be significant (β = 0.25, *p* < 0.01). The SEM also attributed 27% variation to FD and 3% variation to each of the species richness and CWM traits (Figure 3).

**Figure A1:**
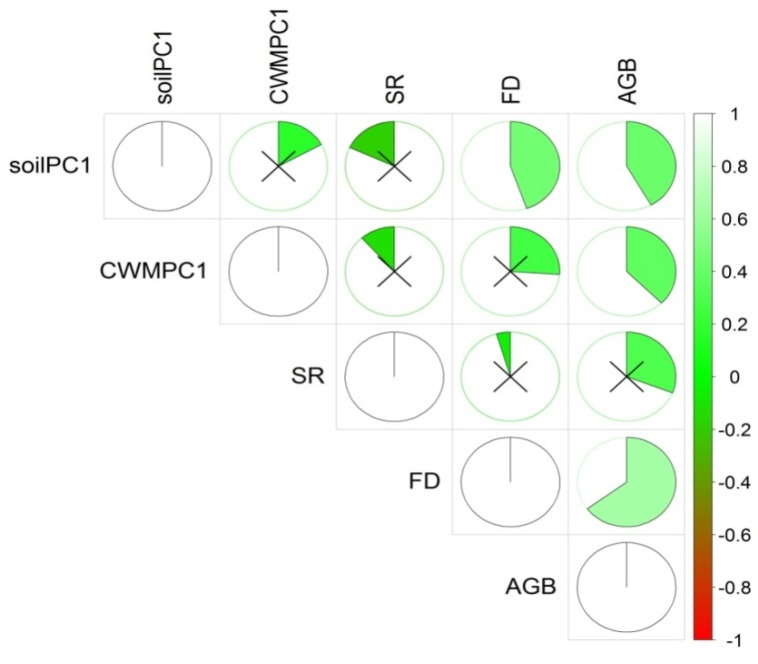
Pearson correlation between the dependent and independent variables used in structural equation modelling (SEM). Aboveground biomass (AGB); Functional diversity (FD); Species richness (SR); Community weighted mean (CWM). Significance *p* < 0.05.

**Figure A2:**
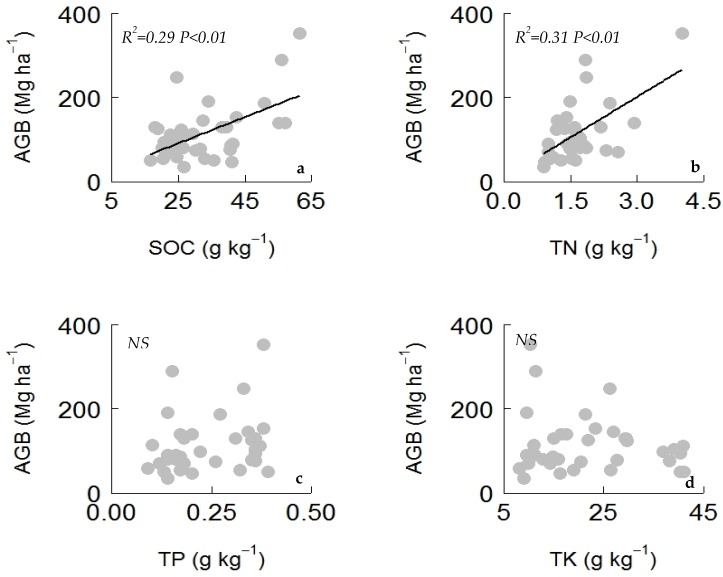
Bivariate relationship between aboveground biomass (dependent variable) and (**a**) soc, (**b**) TN, (**c**) TP and (**d**) (TK) as independent variable. The black line represents fitted linear regression. Significant *p* < 0.05 and nonsignificant (NS) *p* > 0.05.

Soil nutrients had a significant direct positive relationship with FD (β = 0.52, *p* < 0.001) (Table 1), but the relationship was nonsignificant between soil nutrients, species richness, and CWM traits (Figure A1). SOC and TN had a significant relationship with aboveground biomass, while the relationship of TP and TK with aboveground biomass was found to be nonsignificant (Figure A2). The AGB and TN relationship was influenced by the plantation type, while other bivariate relationships was not influenced by plantation types (Table A2).

**Figure 4:**
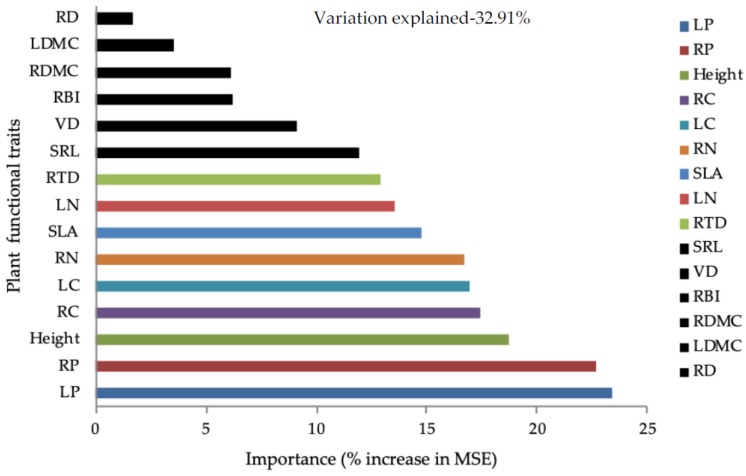
Random forest analysis identifying the best plant functional traits that drive above ground biomass. This analysis identified the important (%increase in Mean Square Error) plant functional traits that drive above ground biomass in the restored forest ecosystem. Bars with different color represent statistically significant predictors (*p* < 0.05) and bars with the same (black) color represent non-significant prediction (*p* > 0.05). The full analysis results are shown in Table A3. Elaboration of the acronyms used in the figure is as follows: LP—leaf phosphorus content; RP—root phosphorus content; RC—root carbon content; LC—leaf carbon content; RN—root nitrogen content; SLA—specific leaf area; LN—leaf nitrogen content; RTD—root tissue density; SRL—specific root length; VD—leaf vein density; RBI—root branching intensity; RDMC—root dry matter content; LDMC—leaf dry matter content; RD—root diameter.

**Figure 5:**
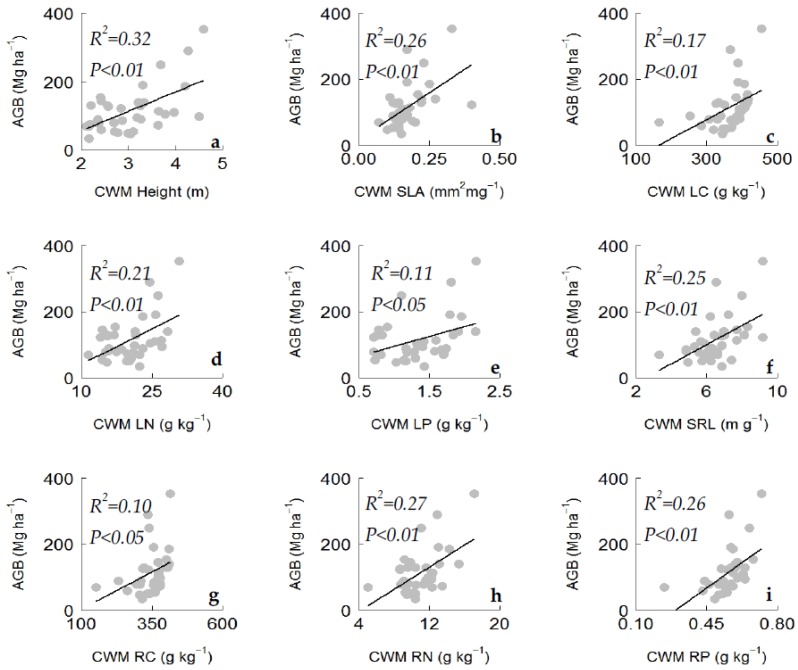
Bivariate relationship between above ground biomass (dependent variable) and selected plant functional traits (independent variable) identified from random forest analysis. Selected significant relationships are shown here. (**a**–**i**) Above ground biomass with (**a**) maximum plant height; (**b**) community weighted mean of specific leaf area (SLA); (**c**)community weighted mean of leaf carbon content (CWM LC); (**d**) community weighted mean of leaf nitrogen content (CWM LN); (**e**) community weighted mean of leaf phosphorus content (CWM LP); (**f**) community weighted mean of specific root length; (**g**) community weighted mean of root carbon content (CWM RC); (**h**) community weighted mean of root nitrogen content (CWM RN); (**i**) community weighted mean of root phosphorus content (CWM RP).

Random forest modelling was applied to identify the most important plant functional traits that contribute in predicting variations in aboveground biomass. The random forest model explained 33% of the variability in aboveground biomass (Table A3). Plant functional traits related to carbon (leaf carbon content, LC; root carbon content, RC), nitrogen (LN, RN), and phosphorus (leaf phosphorus content, LP; root phosphorus content, RP) were found to be the dominant predictors of aboveground biomass (Figure 4). Plant height, SLA, and root tissue density (RTD) were also found to be among the most important predictors of aboveground biomass (Figure 4). We also tested the bivariate relationships between aboveground biomass and the significant plant traits identified by random forest analysis. We found that the plant traits were significantly and positively related to aboveground biomass (Figure 5). The ANCOVA results showed that plantation type had an influence on the AGB–LN and AGB–RN relationships (Table A2), while other bivariate relations was not influenced by plantation types. The results of the bivariate relationships and PCA indicated the existence of a leaf and root economic spectrum with an acquisitive resource use strategy (Table A1).

The correlation analysis showed that SOC was positively (*p* < 0.05) correlated with the CWM of the plant height, SLA, LC, root dry matter content (RDMC), and RC (Table A4), while TN was positively and significantly (*p* < 0.05) correlated with plant height, SLA, LDMC, LN, RDMC, and RN (Table A6). TP was positively (*p* < 0.05) correlated with specific root length (SRL), RP, RTD, root branching intensity (RBI) and negatively correlated with LP (Table A4).

## 3. Discussion

Understanding how biodiversity attributes determine ecosystem functioning (aboveground biomass) is one of the central goals of ecology. This empirical study focused on the relative importance of taxonomic (species richness) diversity and functional diversity in regulating aboveground biomass. Consistent with our hypothesis, the aboveground biomass was found to have a positive relationship with taxonomic and functional diversity (Figure 2). The results from OLS regression and SEM analysis revealed that FD had relatively more explanatory power in predicting variation in the aboveground biomass than did TD in the restored forest ecosystem (Figure 3). Higher FD might provide lower niche overlap among the constituent species and ensure maximum utilization of the available resources which, in turn, can positively influence the aboveground biomass [28]. The dominant species and their functional traits (mass-ratio hypothesis) also increased aboveground biomass. The positive relationship of aboveground biomass with CWM and FD indicates that increased aboveground biomass in the restored forest is driven by the dominant species with rapid acquisitive strategies; and diverse species with diversified nutrient use strategies [1,12]. Therefore, aboveground biomass in the restored forest is regulated by both the mass-ratio hypothesis and the niche complementarity hypothesis (Figure 3). The findings of this study—that aboveground biomass in the restored forest is jointly regulated by the mass-ratio and niche complementarity mechanisms—are consistent with the findings of Ali et al. [12] but contrast with empirical studies that did not find support for either of the two mechanisms [14,29]. Moreover, species richness might have increased aboveground biomass through greater resource capture and efficient utilization of the available resources [9,30]. Additionally, diversity of resources due to diverse species richness across plantation types might have influenced aboveground biomass. Consistent with our findings, species richness was also previously found to have a positive link with aboveground biomass in a subtropical forest [6]. However, some empirical studies found that species richness had a weak or negative relationship with aboveground biomass in sites where the heterogeneity of environmental factors was dominant [1,3]. These results, that plant functional diversity better explained the aboveground biomass than did species richness, are in accordance with previous studies on secondary forests and grassland [18,31].

Plant functional traits that enhance acquisition, sustain plant growth, and allow proficient use of natural resources (solar radiation) and nutrients are essential for sustainable increases in aboveground biomass [20,32]. In our study, plant functional traits related to growth and nutrient acquisition and utilization were found to be the dominant predictors of aboveground biomass. The PCA results revealed that the first PCA axis was driven by CWM traits related to morphology and elemental concentration in plant tissues, representing a community-level leaf and root economic spectrum. These results also suggest that at the community level, plant traits associated with construction cost (leaf and root) and resource acquisition potential might covary, highlighting the presence of an economic spectrum (leaf and root) in the restored forest landscape. Moreover, the PCA loadings and bivariate relationships suggest an acquisitive strategy that tends to influence aboveground biomass. The increase in aboveground biomass with increasing plant height might be due to the presence of fast-growing tall species that are proactive in their efficient utilization of solar radiation. A positive link between aboveground biomass and plant height was previously reported in several empirical studies [1,2,12,29]. The positive relationship between CWM SLA and aboveground biomass increase might be related to the leaf economic spectrum [15], the exploitative species with high SLA facilitate rapid nutrient acquisition and turnover, which might enhance rapid growth and maximize aboveground biomass [24]. Indeed, the CWM of SLA following the carbon gain concept [33] might facilitate higher carbon accumulation; the positive effect of SLA on stand level productivity was also found in tropical rainforests of Australia [34]. The presence of a maximum number of unshaded leaves (e.g., in *Eucalyptus* and coniferous vegetation) is another probable reason for the positive relationship between aboveground biomass and CWM SLA. Therefore, high aboveground biomass in forest communities might be associated with a high proportion of unshaded leaves [12]. Furthermore, the significant increase in aboveground biomass with increasing CWM LC, CWM LN, and CWM LP also suggests that short lived leaves (high SLA), having a higher leaf elemental concentration through their canopy properties, might improve soil properties by adding nutrients into the soil, which, in turn, might influence aboveground biomass [11]. These findings are in agreement with previous empirical studies in a tropical forest [29] and successional biomes of temperate [8] and tropical regions [12], which implies that the presence of a leaf economic spectrum [24] will have a significant impact on canopy properties and ecosystem processes.

Our results also found that high aboveground biomass is associated with root traits. The positive associations of CWM SRL and CWM RN with aboveground biomass indicate a root economic spectrum [26]. SRL and RN have a strong effect on nitrification and have linkage with the plant nitrogen cycling [27]. The rapid acquisition and turnover of fine roots can enhance nutrient cycling which might facilitate plant growth and increased aboveground biomass. The positive association among CWM RN and aboveground biomass might be due to the leguminous species (e.g., the *Acacia mangium* in AM plantation) which fixes atmospheric nitrogen and added to the soil. Moreover, phosphorus is considered to be the most limiting nutrient in the tropical forest ecosystem [30]; thus, an increase in CWM RP might increase phosphorus cycling and more available phosphorus might facilitate plant growth and increase the aboveground biomass of the restored forest community. Therefore, high aboveground biomass in the restored forest is scaled up by the economic spectra and the acquisitive strategies in the leaf and root traits. Moreover, the SEM did not find a significant direct effect of soil nutrients on aboveground biomass, but the bivariate relationship found a positive effect of SOC and TN on aboveground biomass (Figure A2). These results indicate that an increase in SOC and TN during forest restoration through plant-mediated inputs might positively (Table A2) influence plant growth and aboveground biomass [20,35]. FD had a positive relationship with soil nutrients, indicating that soil properties (mainly SOC and TN as suggested by the PC1 loadings) favor a higher dispersion of functional traits that might facilitate the germination of dispersed species or survival of planted/dispersed species. Moreover, SOC and TN were positively correlated with CWM traits (e.g., SLA, LC, LN, RC, RN), suggesting that the dominant species of the community have higher carbon and nitrogen use efficiency [36]. Fast-growing species possessing acquisitive traits might facilitate litter (above and belowground) decomposition and rapid turnover to the soil which in turn might significantly influence the aboveground biomass [32]. Therefore, the greater nutrient retention and resource use capacity might support plant growth and aboveground biomass [37].

## 4. Materials and Methods

### 4.1. Study area and Experimental Forests

This study was conducted at the Heshan National Field Research Station of Forest Ecosystem (112°50′ E and 22°34′ N), Heshan city, Guangdong Province, southern China. A hot and humid climate prevails in the study area, with a mean temperature of 21.7 °C and, mean annual rainfall of 1700 mm. In the study area, April–September is the rainy season and October–March is the dry season. The soil of the region is an ultisol developed from sandstone [38]. Previously, the study area was severely damaged, overexploited, and denuded, causing severe land degradation. Restoration strategies were initiated in 1984 to conserve the degraded hills by plantations across small patches of the forest area. A total area of 12.22 hectares was restored with four plantation types. Among the plantations, three were restored by planting exotic species, and one plantation was restored by planting native species. The plantation types included, 1) a monoculture of *Acacia mangium* (AM); 2) a mixed plantation of Eucalyptus species (*E. exserta*, *E. citriodora*, and *E. camaldulensis*) (EE); 3) a mixed plantation of coniferous species (*Cunninghamia lanceolate* and *Pinus massoniana*) (MC); and 4) a mixed native species plantation (*Schima superba* and *S. wallichii*) (NS). Healthy one-year-old saplings were planted at a 2.5 m × 2.5 m spacing, and the forest was allowed to grow naturally by prohibiting anthropogenic activities around the forest area.

### 4.2. Forest Inventory and Estimation of Aboveground Biomass

**Figure A3:**
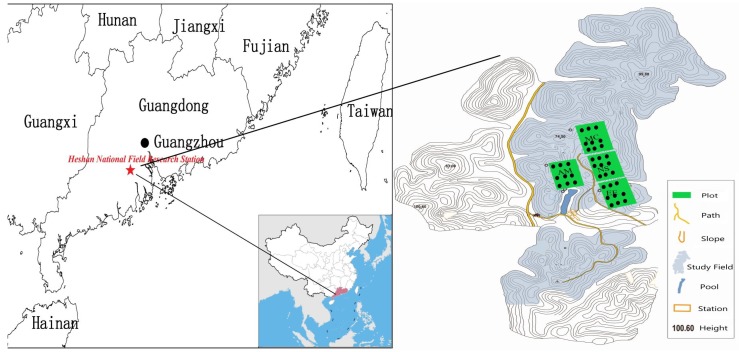
A map of the total study area and the plots selected for this study. The four plantations were located side by side and the plots (black dots) were placed within each plantation types. (AM—*Acacia mangium* plantation, EE—*Eucalyptus* species plantation; MC—mixed coniferous species plantation; NS—native species plantation).

A forest inventory was undertaken at three slope positions (upper slope, middle slope, and lower slope) in each plantation during July–August, 2017 (Figure A3). There were three plots (10 m × 10 m) at each slope position and 36 plots for all the four plantations. In every plot, the height of the trees was measured using either a telescopic pole or a clinometer based on the height of the tree. Diameter at breast height (DBH) was measured for individuals taller than 1.50 m, and the diameter at mid-height or 45cm height was measured for individuals <1.50m tall. Subsequently, we used allometric equation based on the height (H, m) and DBH (cm) to calculate the aboveground biomass of the trees following Chen et al. [39]:

Aboveground biomass = a × (DBH^2^ × H) ^b^ (1)

where a and b are statistical parameters; values of the statistical parameters and details of the equations are presented in Table A5.

### 4.3. Soil Sampling and Soil Chemical Analysis

Within each plot, surface soil samples at the depth of 0–20 cm were collected using a soil auger. We randomly collected 108 soil cores from 36 plots, three soil cores from each plot. To prevent cross-contamination, the soil auger was cleaned and sterilized (70% ethanol) properly between each soil sample collection. After sampling, soils were sieved (2 mm mesh) and air dried for chemical analysis. Soil total carbon (SOC), total nitrogen (TN), total phosphorus (TP), and total potassium (TK) were determined in this study. An Elementar analyzer (Vario elemental analyzer, Langenselbold, Germany) was used to determine the SOC and TN. The molybdenum blue method followed by colorimetric analysis [40] was applied to measure TP, and extraction with 1M NH_4_OAc followed by spectrophotometry with an atomic absorption flame spectrophotometer was used to determine the TK [41].

### 4.4. Plant Functional Traits

The plant functional traits of dominant species were measured based on the standard protocols described by Cornelissen et al. [42]. A total of 20 dominant species was selected based on their relative abundance and some species were found to be common across the four plantation types (Table A6). The plant functional traits measured in this study were as follows: maximum plant height; specific leaf area (SLA); leaf dry matter content (LDMC); leaf vein density (VD); leaf carbon, nitrogen, and phosphorus (LC, LN, and LP, respectively); root diameter (RD); specific root length (SRL); root dry matter content (RDMC); root tissue density (RTD); root branching intensity (RBI); and root carbon, nitrogen, and phosphorus (RC, RN, and RP, respectively). The above- and below-ground functional traits were measured from 5 individuals and 25 samples of leaves and roots were collected for each species. Leaves fully exposed to sunlight were collected to measure the SLA and LDMC. A leaf area meter (Li-Cor 3100C Area Meter, Li-Cor, Lincoln, USA) was used to measure the leaf area of fresh and turgid leaves. Leaves were then oven dried at 60 °C for 72 hours [29]. We calculated the SLA by dividing the leaf area by its dry mass; LDMC was measured by dividing the fresh mass by its dry mass. The dried leaves were ground in a ball mill and sieved to determine the total nitrogen and total carbon using an Elementar analyzer (Vario Micro Cube elemental analyzer, Langenselbold, Germany). Leaf vein density was measured by the standard protocol described by Peìrez-Harguindeguy et al. [43]. At the base of the trees, a specially constructed fork was used to excavate the surface soil, and fine roots that were attached to the main lateral roots were collected for this study. The depth of the soil excavation was different based on the species. Roots were washed with deionized water, stored in an icebox and transported to the laboratory within 4 hours. An Epson Expression 10000XL flatbed scanner and image processing software WINRHIZO (Regent Instruments Inc., Sainte-Foy Sillery-Cap-Rouge, Canada) were used to obtain the root length, root diameter, and root volume. The roots were scanned at 300dpi [43]. Furthermore, the roots were oven dried to constant weight; SRL was measured as the ratio of root length to its dry mass and RDMC was determined by the fresh mass of root with its dry mass. Again, the dried ground powder of roots obtained from a ball mill was used to measure the root total carbon and nitrogen content using an Elementar (Vario Micro Cube elemental analyzer, Langenselbold, Germany). A similar method was applied for leaf and root phosphorus estimation as was used for soil phosphorus. The RTD was measured by dividing the root dry mass with its volume and the RBI was measured by dividing the number of root tips by the total root length [44]. 

We measured the CWM for each trait; the CWM is the abundance-weighted mean trait value for a community and was calculated with the following formula:

CWM (trait_x_) = ΣP_i_T_i_ (2)

where, P_i_ is the relative abundance for the i^th^ species in the community and T_i_ is the mean trait value of the i^th^ species in the community. The single trait functional dispersion in each forest community [45] was measured by using the formula:

(3) $$\mathrm{FD}=\overset{n}{\underset{i=1}{\sum }}p_{i}\frac{\left|T_{i}-CWT_{i}\right|}{\sum_{i}^{n}\left|T_{i}-CWT_{i}\right|}$$

Further, FD_is_ was measured including all traits following Laliberté and Legendre [46]:

FD_is_= ∑ (P_ij_Z_j_) / ∑ A_j_ (4)

c= ∑ (P_ij_T_jj_) / ∑ A_j_ (5)

where T_ij_ is the value of trait i for species j, Aj is the abundance of species j, and c is used to calculate Z_j_, the distance of species j to the weighted centroid.

The values of the CWM of each trait and characteristics of other variables are presented in Table A7.

### 4.5. Statistical Analysis

To test the relationship between aboveground biomass and plant diversity metrics (taxonomic and functional diversity), we constructed an ordinary least squares (OLS) linear regression model. Again, we used structural equation modelling (SEM) to determine the relationships of soil nutrients, plant diversity metrics (taxonomic diversity, functional diversity), and CWM traits with the ecosystem aboveground biomass. A hypothesized meta-model was constructed based on the predictor variables (Figure 1). The soil chemical properties (SOC, TN, TP, and TK) were converted into a single variable through PCA, and the first PC of soil properties described 66% of the variation. The same analysis was also conducted to convert the leaf and root CWM into a single variable. The PC1 of CWM traits described 91% of the variation (Table A2). We have picked PC1 for both CWM traits and soil properties for further analysis based on the parallel test (Table A8) performed in R [47]. To fit the model, the maximum-likelihood estimation method was applied. Model fitness was checked through non-significant Chi-square (χ2) test statistics (*p* > 0.05), low standardized root mean square residual (SRMR < 0.08), and a high goodness of fit index (GFI > 0.95) and comparative fit index (CFI). Direct and indirect pathways among the variables were assessed to identify the net influence of one variable on another. Standardized direct and indirect effects were added to calculate the total effect [12]. This analysis was conducted using the lavaan package [48] of R 3.2.2 [47]. Furthermore, random forest modelling was applied to detect the most important plant functional traits that influence aboveground biomass. Random forest modelling does not depend on a single regression; rather, it aggregates multiple classification trees through bootstrap aggregation. The importance of each predictor was determined by the increase in the mean square error (MSE) and was averaged over 5000 trees. This analysis was performed using the rfPermute package in [49] in R. Moreover, the plant traits identified by random forest analysis were tested through bivariate regression analysis where the aboveground biomass was dependent variable and the CWM traits were the independent variable. We did ANCOVA test to determine the effect of plantation type on the bivariate relationships between AGB with other predicting variables. Moreover, analysis of variance (ANOVA) test was performed and revealed, AGB and other predicting variables were not significantly influenced by the slopes of the hill (Table A9). Finally, correlation (Pearson correlation) analysis between soil properties and CWM plant traits was performed to determine the influence of soil properties on plant functional traits.

## 5. Conclusions

We conclude that, in assessing the relative contributions of plant taxonomic (species richness) and functional diversity for predicting aboveground biomass, functional diversity explains more than the taxonomic diversity. We found that the mass-ratio hypothesis and the niche complementarity hypothesis were not mutually exclusive; rather, these metrics jointly predicted the variability in aboveground biomass [11]. However, FD following the niche complementarity hypothesis was more important than the CWM (i.e.,the mass ratio hypothesis). These results indicate that efforts should be made to maintain high numbers of standing dominant species having diverse functional traits to facilitate aboveground biomass in restored forest landscapes. The results of this study also suggest the existence of leaf and root economic spectrum. Therefore, the inclusion of root functional traits along with leaf functional traits might improve our understanding of the contribution of functional diversity in explaining the variation in aboveground biomass. The plant functional traits related to rapid nutrient acquisition and nutrient turnover were found to be the dominant predictors of aboveground biomass. Overall, this study enriched our understanding of the significant biodiversity metrics and plant functional traits that influence ecosystem functioning (aboveground biomass) and can guide successful management of forests in tropical and subtropical regions. Future studies on the relationship between aboveground biomass and biodiversity metrics might focus on forests distributed across a large scale and exposed to contrasting environmental gradients.

## Figures and Tables

**Table 1:** The direct, indirect, and total standardized effects of soil nutrients, functional diversity (FD), species richness, and community weighted means (CWM) on aboveground biomass (AGB) across subtropical forest of southern China based on structural equation models (SEMs). Significance at (*p* < 0.05).

Predictor	Pathway to Response Variable	Response Variable	Effect	*P*-Value
Soil nutrient	Direct effect	AGB	0.20	0.117
	Direct effect	FD	0.52	<0.001
	Direct effect	SR	−0.18	0.269
	Direct effect	CWM	0.17	0.304
	Indirect effect via FD	AGB	0.25	0.008
	Indirect effect via SR	AGB	−0.07	0.290
	Indirect effect via CWM	AGB	0.04	0.345
	Total effect	AGB	0.43	0.004
	Total effect	FD	0.52	<0.001
	Total effect	SR	−0.18	0.269
	Total effect	CWM	0.17	0.304
FD	Direct effect	AGB	0.49	<0.001
	Total effect	AGB	0.49	<0.001
Species richness	Direct effect	AGB	0.40	<0.001
	Total effect	AGB	0.40	<0.001
CWM	Direct effect	AGB	0.26	0.018
	Total effect	AGB	0.26	0.018

AGB—aboveground biomass; FD—functional diversity; SR—species richness; CWM—community weighted mean.

## Appendix A

**Table A1:** Eigenvalues and variances for the first two components in Principal Components Analysis (PCA) of leaf and root functional traits.

Soil PCA			CWM PCA		
Variables	PC 1 (66.05%)	PC 2 (33.82%)	Variables	PC 1 (91.44%)	PC 2 (6.71%)
SOC	0.82074	0.57086	HEIGHT	0.00721	−0.003658
TN	0.018358	0.012611	SLA	0.000370	−0.000316
TP	−0.00375	0.005537	LDMC	0.40724	0.32336
TK	−0.57099	0.82093	VD	0.00065	−0.006267
			LC	0.55928	−0.46773
			LN	0.08116	−0.023379
			LP	0.002254	0.000754
			RD	0.000929	0.000469
			SRL	0.03066	−0.02578
			RDMC	0.4403	0.74325
			RTD	0.000944	0.000286
			RBI	0.006874	−0.00497
			RC	0.57147	−0.34109
			RN	0.0239	−0.016097
			RP	0.000761	−0.00139

SOC, TN, TP, TK values were used to get PCA scores of soil and all the traits measured were used to obtain CWM PCA.

**Table A2:** Analysis of covariance (ANCOVA) test showing significance levels for the slopes of the regression equation for each pair of variables among the four plantations. Significance at (*p* < 0.05).

Pair of Variables	P-Value (Continuous Predictor)	P-Value (Plantation Type)	P-Value (Slope Position)	P-Value (Interaction Effect)
AGB-TD	<0.001	0.015	0.615	0.174
AGB-FD	<0.001	0.163	0.708	0.268
AGB-CWM	0.04	0.755	0.890	0.442
AGB-SOC	<0.001	0.138	0.272	0.750
AGB-TN	<0.001	0.046	0.602	0.608
AGB-Height	<0.001	0.432	0.920	0.713
AGB-SLA	0.003	0.527	0.666	0.534
AGB-LC	0.007	0.885	0.856	0.764
AGB-LN	<0.001	0.254	0.665	0.963
AGB-LP	<0.001	<0.001	0.934	0.136
AGB-SRL	<0.001	0.109	0.882	0.069
AGB-RC	0.03	0.0838	0.932	0.397
AGB-RN	<0.001	0.034	0.954	0.253
AGB-RP	<0.001	0.471	0.907	0.894

Each row represents a separate model, AGB is the response variable and other variables are the continuous variable. Elaboration of the acronyms has been presented in Figure 2 and Figure 5.

**Table A3:** Random forest modelling for identifying important predictors explaining above ground biomass in restored forest of southern China. Importance of the predictors was measured by % increase in mean square of error (MSE). *p*-value identifies the significant predictors.

Predictors	Variation Explained (32.91%)	
	%Increase in MSE	*p*-Value
LP	23.38	0.01
RP	22.68	0.03
Height	18.73	0.01
RC	17.44	0.03
LC	16.89	0.03
RN	16.69	0.04
SLA	14.78	0.05
LN	13.52	0.03
RTD	12.92	0.05
SRL	11.91	0.25
VD	9.09	0.49
RBI	6.211	0.21
RDMC	6.12	0.43
LDMC	3.55	0.67
RD	1.66	0.70

Elaboration of the acronyms has been presented in Figure 5.

**Table A4:** Correlation coefficients (Pearson correlation) of soil properties and above and below ground community weighted mean (CWM) traits of the restored forest ecosystem.

	HEIGHT	SLA	LDMC	LC	LN	LP	SRL	RDMC	RTD	RBI	RC	RN	RP
SOC	0.350 *	0.389 *	0.281	0.365 *	0.304	0.513 **	0.135	0.328 *	0.14	0.122	0.359 *	0.519 **	0.267
TN	0.516 **	0.506 **	0.493 **	0.531 **	0.470 **	0.635 **	0.156	0.443 **	0.22	−0.08	0.537 **	0.721 **	0.464 **
TP	0.148	0.209	0.158	0.31	0.003	−0.363 *	0.353 *	−0.03	0.443 **	0.420 **	0.315	−0.017	0.509 **
TK	0.09	−0.05	0.14	0.22	−0.09	0.157	0.065	0.003	0.446 **	0.351 *	0.254	−0.094	0.365 *

Significance at * *p* < 0.05; ** *p* < 0.01; Elaboration of the acronyms has been presented in Figure 4 and Figure 5.

**Table A5:** The allometric equations used to calculate the aboveground biomass of species across the restored forest ecosystem.

	Aboveground Biomass
Species	Allometric Equation
*Acacia mangium*	0.1751 × (DBH^2^ × H)^0.7466^
*Cinnamomum burmannii*	0.0087 × (DBH^2^ × H)^1.0323^
*Clerodendrum fortunatum*	1.6461 × (DBH^2^ × H)^0.6825^
*Cunninghamia lanceolata*	0.0087 × (DBH^2^ × H)^1.0323^
*Eucalyptus exserta*	0.0811 × (DBH^2^ × H)^0.8264^
*Eucalyptus rostrata*	0.0811 × (DBH^2^ × H)^0.8264^
*Eucalyptus urophylla*	0.0811 × (DBH^2^ × H)^0.8264^
*Eurya chinensis*	0.5092 × (DBH^2^ × H)^0.9403^
*Gardenia jasminoides*	0.8948 × (DBH^2^ × H)^0.7417^
*Ilex asprella*	7.7741 × (DBH^2^ × H)^0.5995^
*Litsea cubeba*	0.5423 × (DBH^2^ × H)^0.8742^
*Litsea glutinosa*	0.4868 × (DBH^2^ × H)^0.9218^
*Litsea rotundifolia*	0.0423 × (DBH^2^ × H)^0.8949^
*Melastoma malabathricum*	0.2475 × (DBH^2^ × H)^1.0904^
*Melicope pteleifolia*	0.8246 × (DBH^2^ × H)^0.7595^
*Pinus massoniana*	0.0156 × (DBH^2^ × H)^1.0359^
*Psychotria asiatica*	0.4596 × (DBH^2^ × H)^0.9482^
*Rhodomyrtus tomentosa*	0.0506 × (DBH^2^ × H)^1.4907^
*Schima superba*	0.0629 × (DBH^2^ × H)^0.4643^
*Schima wallichii*	0.0629 × (DBH^2^ × H)^0.4643^

**Table A6:** List of species selected for this study along with their characteristics across four plantation forests.

Species	Family	Growth Form	Plant Type	Nitrogen Fixation
**AM**				
*Acacia mangium*	Fabaceae	Tree	Evergreen	Nitrogen fixing
*Cinnamomum burmannii*	Lauraceae	Tree	Evergreen	Non-nitrogen fixing
*Eurya chinensis*	Theaceae	Tree	Evergreen	Non-nitrogen fixing
*Ilex asprella **	Aquifoliaceae	Tree	Deciduous	Non-nitrogen fixing
*Litsea cubeba*	Lauraceae	Tree	Evergreen	Non-nitrogen fixing
*Litsea rotundifolia*	Lauraceae	Tree	Evergreen	Non-nitrogen fixing
*Melicope pteleifolia **	Rutaceae	Tree	Deciduous	Non-nitrogen fixing
*Rhodomyrtus tomentosa **	Myrtaceae	Tree	Evergreen	Non-nitrogen fixing
*Clerodendrum fortunatum **	Lamiaceae	Shrub	Evergreen	Non-nitrogen fixing
*Gardenia jasminoides**	Rubiaceae	Shrub	Evergreen	Non-nitrogen fixing
*Litsea glutinosa*	Lauraceae	Shrub	Evergreen	Non-nitrogen fixing
**EE**				
*Eucalyptus exserta*	Myrtaceae	Tree	Evergreen	Non-nitrogen fixing
*Eucalyptus rostrata*	Myrtaceae	Tree	Evergreen	Non-nitrogen fixing
*Eucalyptus urophylla*	Myrtaceae	Tree	Evergreen	Non-nitrogen fixing
*Ilex asprella **	Aquifoliaceae	Tree	Deciduous	Non-nitrogen fixing
*Litsea cubeba*	Lauraceae	Tree	Evergreen	Non-nitrogen fixing
*Litsea glutinosa*	Lauraceae	Tree	Evergreen	Non-nitrogen fixing
*Melicope pteleifolia **	Rutaceae	Tree	Deciduous	Non-nitrogen fixing
*Clerodendrum fortunatum **	Lamiaceae	Shrub	Evergreen	Non-nitrogen fixing
*Gardenia jasminoides **	Rubiaceae	Shrub	Evergreen	Non-nitrogen fixing
*Melastoma malabathricum*	Melastomataceae	Shrub	Evergreen	Non-nitrogen fixing
*Rhodomyrtus tomentosa **	Myrtaceae	Shrub	Evergreen	Non-nitrogen fixing
**MC**				
*Cunninghamia lanceolata*	Pinaceae	Tree	Evergreen	Non-nitrogen fixing
*Ilex asprella **	Aquifoliaceae	Tree	Deciduous	Non-nitrogen fixing
*Melicope pteleifolia **	Rutaceae	Tree	Deciduous	Non-nitrogen fixing
*Pinus massoniana*	Pinaceae	Tree	Evergreen	Non-nitrogen fixing
*Schima superba*	Theaceae	Tree	Evergreen	Non-nitrogen fixing
*Rhodomyrtus tomentosa **	Myrtaceae	Tree	Evergreen	Non-nitrogen fixing
*Clerodendrum fortunatum **	Lamiaceae	Shrub	Evergreen	Non-nitrogen fixing
*Gardenia jasminoides **	Rubiaceae	Shrub	Evergreen	Non-nitrogen fixing
*Psychotria asiatica*	Rubiaceae	Shrub	Evergreen	Non-nitrogen fixing
**NS**				
*Ilex asprella **	Aquifoliaceae	Tree	Deciduous	Non-nitrogen fixing
*Melicope pteleifolia **	Rutaceae	Tree	Deciduous	Non-nitrogen fixing
*Psychotria asiatica*	Rubiaceae	Tree	Evergreen	Non-nitrogen fixing
*Schima wallichii*	Theaceae	Tree	Evergreen	Non-nitrogen fixing
*Schima superba*	Theaceae	Tree	Evergreen	Non-nitrogen fixing
*Clerodendrum fortunatum **	Lamiaceae	Shrub	Evergreen	Non-nitrogen fixing
*Eurya chinensis*	Theaceae	Shrub	Evergreen	Non-nitrogen fixing
*Gardenia jasminoides **	Rubiaceae	Shrub	Evergreen	Non-nitrogen fixing
*Rhodomyrtus tomentosa **	Myrtaceae	Shrub	Evergreen	Non-nitrogen fixing

* Common species; AM—*Acacia mangium* plantation; EE—mixed *Eucalyptus* species plantation; MC—mixed coniferous species plantation; NS—mixed *Schima* species plantation

**Table A7:** Summary of the characteristics (value range, mean, standard error of mean) of variables along with their acronyms and measuring units.

Variables	Acronym	Value Range	Mean ± Standard Error	Units
Aboveground biomass	AGB	34.18-352.31	115.03±11.42	Mg ha^-1^
Species richness	TD	8-21	13.25±0.595	number
Functional diversity	FD	0.06-0.25	0.12±0.007	Unitless
Maximum plant height	Height	2.1-4.59	3.02±0.118	m
Specific leaf area	SLA	0.07-0.4	0.17±0.010	mm^2^ mg^-1^
Leaf dry matter content	LDMC	97.35-298.07	230.30±6.525	mg g^-1^
Leaf vein density	VD	2.11-11.92	4.21±0.261	mm mm^-2^
Leaf carbon content	LC	164.9-454.54	364.59±8.873	g kg^-1^
Leaf nitrogen content	LN	11.38-30.67	20.34±0.784	g kg^-1^
Leaf phosphorus content	LP	0.71-2.16	1.33±0.071	g kg^-1^
Root diameter	RD	0.21-4.09	0.58±0.103	mm
Specific root length	SRL	3.3-9.17	6.49±0.203	m g^-1^
Root dry matter content	RDMC	104.17-344.55	229.59±7.491	mg g^-1^
Root tissue density	RTD	0.18-0.65	0.46±0.019	gcm^-3^
Root branching intensity	RBI	0.5-6.5	3.60±0.240	tipscm^-1^
Root carbon content	RC	151.54-414.79	346.85±8.966	g kg^-1^
Root nitrogen content	RN	5.05-17.07	11.05±0.365	g kg^-1^
Root phosphorus content	RP	0.24-0.72	0.55±0.013	g kg^-1^
Soil organic carbon	SOC	16.51-61.37	32.52±2.025	g kg^-1^
Total nitrogen	TN	0.9-4.01	1.64±0.102	g kg^-1^
Total phosphorus	TP	0.09-0.39	0.24±0.016	g kg^-1^
Total potassium	TK	8.07-41.02	21.96±1.810	g kg^-1^

**Table A8:** Results of Horn’s Parallel Analysis for component retention (Adjusted eigenvalues > 1 indicate dimensions to retain).

Component	Adjusted Eigenvalue	Unadjusted Eigenvalue	Estimated Bias
CWM traits PCA			
1	1.631151	1.256974	0.625823
2	0.663314	0.839614	0.176299
Soil PCA			
1	1.355838	1.743546	0.387708
2	0.327638	0.421766	0.094127

**Table A9:** Analysis of variance (ANOVA) table showing the significance of different variables influenced by the hill slopes (Significance *p* < 0.05).

Variables	Sum of Squares	DF	Mean Square	F	Sig.
AGB	2183315.682	2	1091657.841	2.526244	0.095315
TD	10.16666667	2	5.083333333	0.384234	0.683979
FD	0.008205556	2	0.004102778	2.469524	0.100128
HEIGHT	0.666672222	2	0.333336111	0.651748	0.527712
SLA	0.020816667	2	0.010408333	2.795131	0.075613
LDMC	3150.75905	2	1575.379525	1.029242	0.368462
VD	9.624155556	2	4.812077778	2.081727	0.140788
LC	8581.352106	2	4290.676053	1.562361	0.224753
LN	120.6284667	2	60.31423333	3.03795	0.061515
LP	0.392372222	2	0.196186111	1.056245	0.359222
RD	0.83495	2	0.417475	1.09229	0.347269
SRL	4.315872222	2	2.157936111	1.486274	0.240966
RDMC	3942.490289	2	1971.245144	0.974221	0.38808
RTD	0.016538889	2	0.008269444	0.612208	0.548195
RBI	5.078505556	2	2.539252778	1.236306	0.303559
RC	8132.912822	2	4066.456411	1.440282	0.251363
RN	17.37561667	2	8.687808333	1.897105	0.166004
RP	0.014372222	2	0.007186111	1.051393	0.360864

Elaboration of the acronyms has been presented in Figure 4 and Figure 5.
